# Improving access to healthcare services for sickle cell disease patients in Nigeria: perspectives and views of healthcare professionals

**DOI:** 10.3389/frhs.2025.1466299

**Published:** 2025-08-25

**Authors:** Godspower Onavbavba, Obi Peter Adigwe, Solomon Oloche Onoja

**Affiliations:** ^1^ National Institute for Pharmaceutical Research and Development, Abuja, Nigeria; ^2^ Department of Medical Laboratory Sciences, University of Nigeria, Enugu, Nigeria

**Keywords:** sickle cell disease, haematology, genotype, public health, genetic, care

## Abstract

**Introduction:**

In sub-Saharan Africa, the burden of sickle cell disease remains high. With annual sickle cell births of about 150,000, Nigeria is reported to have the highest prevalence of the disease globally. This study aimed to explore the views and perspectives of healthcare professionals regarding access to healthcare services for sickle cell disease.

**Methods:**

A quantitative cross-sectional design was employed for this study. Participants comprised healthcare practitioners across Nigeria. A well-structured questionnaire was utilised for data collection. A stratified multistage sampling strategy was used for the study, and respondents were recruited from all the six geographical zones in the country. Data collected were entered into Statistical Package for Social Sciences (SPSS) software version 25. Descriptive and inferential statistical analyses were undertaken. Results were presented in frequencies and percentages.

**Results:**

The response rate was 83.5% (1,002/1,200); male and female participants were of similar proportions, as indicated by 51.2% and 48.8%, respectively. A significant proportion of the participants (43.1%) disagreed that development partners have adequately contributed to the funding of sickle cell disease research in Nigeria. The majority of the respondents (81%) indicated that providing special funding for health research can facilitate access to healthcare services for sickle cell patients, whilst a similar proportion (79.2%) disagreed that the government alone bears the responsibility for healthcare initiatives for the disease. A third of the study participants (67.8%) were of the view that current research and development efforts towards sickle cell disease were inadequate.

**Conclusion:**

This study describes health professionals' views on access to healthcare for sickle cell, and the findings revealed the criticality of private and development sector funding in reducing the burden of the disease. Furthermore, capacity building at the primary healthcare level would not only ensure access to the basic healthcare needs of patients but could also demystify the condition.

## Introduction

Sickle cell disease is a severe hematologic disorder that requires robust and timely clinical intervention (1). It is characterised by episodes of pain resulting from vaso-occlusion and progressive organ damage. Sickle cell disease is one of the most common monogenetic diseases worldwide, with approximately 300,000 births annually (2, 3). This disease has been recognised as a global health problem estimated to cause over 376,000 deaths yearly (4). Sickle cell disease requires a continuum of care to achieve optimum quality of life (5), as poor management can result in complications, recurrent health crises, and low life expectancy (6). As evidenced in the literature, the survival rate can be greatly improved by providing adequate high-quality care for those suffering from the disease (5).

The clinical management of the disease in developing countries is grossly lacking, as the condition is still associated with high mortality rates (7). In Africa, approximately 50%–80% of children born with sickle cell disease do not survive beyond their fifth year (8). In contrast, life expectancy in developed countries has significantly improved, with almost all infants now expected to survive into adulthood owing to robust and comprehensive care programs (9). For instance, in the Netherlands, the management of sickle cell disease patients is organised in centralised care centres to ensure good quality of care (10). This underscores the need to address the challenges facing sickle cell management in sub-Saharan Africa, particularly in Nigeria, which bears the largest burden of the disease (11). High prevalence rates of the sickle cell trait have also been reported in countries such as Gabon, the Democratic Republic of Congo, Ghana and Uganda (12–17).

Despite global advancements in sickle cell disease management in the past few decades, Nigeria remains disproportionately affected by the burden of the disease, and the mortality rate is higher in children (6). There has been a significant epidemiologic rise from 30,000 to about 150,000 annual sickle cell births in the last few decades (18, 19); suggesting that the country's health care system has not given critical attention to preventive strategies for the ailment (20).

Healthcare professionals play a central role in the management of sickle cell disease, from early diagnosis to long-term care and crisis prevention. Their knowledge, attitudes, and clinical practices significantly influence patient outcomes, especially in resource-constrained settings like Nigeria. Studies have shown that adequate provider knowledge and engagement can improve adherence to treatment regimens, reduce complications, and enhance quality of life for individuals living with the condition (21, 22). However, gaps in provider training, limited access to continuing education, and poor integration of SCD care into primary healthcare services remain major barriers (23, 24). Therefore, understanding healthcare professionals' perspectives is crucial for identifying context-specific strategies to improve care delivery and patient outcomes.

Available evidence suggests that education level influences the healthcare-seeking behaviours of individuals with sickle cell disease and the occurrence of complications related to the condition. Regions characterised by high illiteracy rates in Nigeria are linked to a greater prevalence of sickle cell disease and unfavourable health outcomes (25). Poverty and low socioeconomic status have also been associated with higher rates of the disease, shorter life expectancy, and several other negative outcomes (21, 26). Furthermore, persons with sickle cell disease have been reported to encounter many psychosocial issues, including increased anxiety, depression, aggression, poor relationships, and impaired health-related quality of life (27, 28).

Given these socioeconomic challenges, it is important to understand how healthcare financing mechanisms in Nigeria influence access to care for sickle cell disease patients. Health care in Nigeria is funded through tax revenues, out-of-pocket payments, health insurance (social, community-based, and private), donor contributions, and exemptions or subsidies. The federal government allocates 3.5%–6.24% of its national budget to health, which is equivalent to 12% of total health financing (29). The majority of the healthcare financing (69%) is shouldered by the patients. This figure is far above the WHO-recommended percentage for out-of-pocket expenditure (20%) (30). State governments, local government areas, and development partners account for 8%, 4%, and 3% of the expenditure, respectively (29) Sickle cell disease care mirrors this overall funding structure where most treatment expenses are paid directly by patients, with only modest supplementation from grants for awareness activities in certain parts of the country (31). As a result, financing dedicated specifically to sickle cell disease represents only a small fraction of overall health spending, compounding existing challenges in accessing high-quality care.

Several studies have underscored the substandard quality of healthcare provided for individuals with sickle cell disease in Nigeria (32–34). However, there is a notable gap in the existing literature regarding the understanding of the specific measures and strategies required to improve both the accessibility and the quality of care for sickle cell patients. Given the critical roles healthcare professionals play in delivering care and their knowledge of the barriers faced by patients, it is important to assess their opinions in this regard. Therefore, this study aimed to explore the perspectives of healthcare practitioners regarding access to healthcare services for sickle cell disease patients in Nigeria. Specifically, it seeks to assess healthcare professionals' perceptions of existing support for sickle cell research and development, evaluate their views on capacity building for primary healthcare workers, determine their opinion on the current extent of research on the disease and gather recommendations from healthcare professionals on how to improve access to high quality healthcare services. The findings are expected to generate actionable insights that can inform decision-making about healthcare services for sickle cell disease patients.

## Methods

A quantitative cross-sectional study was undertaken in Nigeria between October 2021 and June 2022. An English language questionnaire (See Supplementary Material) was utilised for data collection. The tool was designed for the study following an extensive review of existing literature. A panel of individuals involved in research activities in the area of sickle cell disease was used to develop the questionnaire items. All the members of the panel critically reviewed items on the instrument. The questionnaire items were appropriately designed to provide relevant insights into strategies to improve access to healthcare services for sickle cell patients. It covered five key domains: socio-demographic characteristics, perceptions of funding and policy support, views on the role of primary healthcare, opinions on research and development efforts, and perceived barriers and proposed strategies for enhancing access to care, including a question on phytotherapy. This item was included to reflect the cultural relevance and common use of phytomedicines in the management of sickle cell symptoms in Nigeria. The face and content validations of the questionnaire were undertaken using an independent expert panel comprising six members. Panel members were selected based on the following criteria: a minimum of 5 years of experience in sickle cell disease research or clinical management; professional backgrounds spanning public health, haematology, pharmacology, and health systems; demonstrated involvement in policy or research related to sickle cell disease; and representation from different geopolitical zones in Nigeria to ensure contextual relevance. The panel reviewed each item for clarity, relevance, and cultural appropriateness. Revisions were made based on their feedback until content validity thresholds were met. The study tool was assessed for relevant attributes required for it, and some of the statements were rephrased to appropriately suit the context. The content validity ratio and index were tested for each item, and only those that passed these tests were included in the final questionnaire. A pilot testing of the questionnaire was undertaken by administering it to an initial cohort of 20 healthcare practitioners, and no major change was required due to the feedback obtained.

A minimum sample size of 1,066 was calculated for an estimated number of 0.9 million healthcare professionals in Nigeria. This was computed at 95% confidence level, 3% margin of error, and 50% response distribution using Epi Info software version 7. The sample was rounded up to 1,200. A stratified multistage sampling strategy was employed to recruit participants for this study. The process involved first; stratifying Nigeria into its six geopolitical zones. Then, randomly selecting one state from each zone, including the FCT. The states selected were, Adamawa (North East), Lagos (South West), Delta (South South), Anambra (South East) and Kano (North West). FCT represented the North Central region. Within each chosen state, four healthcare facilities managing sickle cell disease were randomly selected. This was to balance national representativeness with logistical feasibility. This number allowed for variation across facility types and urban-rural locations, whilst staying within the constraints of time, personnel, and budget. Within each facility, a roster of eligible practitioners was obtained and participants randomly selected until the facility quota (∼50) was met, yielding 1,002 completed surveys. This ensures representation across zones. To enhance the methodological rigor of the study, these procedures, including stratified sampling, random selection, and the use of a validated questionnaire, were implemented to improve representativeness and minimise potential selection and information bias.

Prior to the collection of data, ethical clearance request was submitted to the Federal Capital Territory Health Research Ethics Committee. Approval was received before the commencement of data collection (Approval number, FHREC/2021/01/97/12-08-21). Participation in the study was voluntary, and written informed consent was obtained from participants prior to the administration of the questionnaires. Absolute confidentiality was ensured during the data collection process.

The inclusion criteria for the study include healthcare professionals who were practising in Nigeria, willing to participate in the study, and working in a hospital setting that manages sickle cell disease. Respondents who did not meet the stated criteria were excluded from participating in the study. Paper-based questionnaires were administered to doctors, pharmacists, medical laboratory scientists, nurses, and other healthcare workers in several healthcare facilities that were visited.

Following the importation of data collected into Statistical Package for Social Sciences software version 25, descriptive statistical analysis was performed. In addition, Chi-square tests were used to examine associations between key demographic variables and selected outcome variables. These outcome variables were identified based on their relevance to the study objectives and the anticipated policy significance of healthcare professionals' perspectives. Specifically, items related to health research funding, the role of government in healthcare provision, and strategies for improving access to care were prioritised. The selection of these variables was informed by existing literature and expert input during questionnaire development. A *p*-value of 0.05 or less was considered the threshold for statistical significance.

## Results

### Socio-demographic data

Of the 1,200 questionnaires distributed across different health facilities, 1,002 were completed and returned, giving a response rate of 83.5%. Of the respondents, 51.2% were male and 48.8% were female. The majority of the study participants (56.3%) were aged 30 years and below, whilst those above 50 years (1.7%) represented the least proportion. Further details regarding the socio-demographic characteristics are presented in Table 1.

**Table 1:** Socio demographic characteristics.

Variable	Frequency (%)
Gender	
Male	513 (51.2)
Female	489 (48.8)
Age (years)	
≤30	564 (56.3)
31–40	285 (28.4)
41–50	118 (11.8)
51 and above	17 (1.7)
Missing	18 (1.8)
Highest educational level	
National diploma	284 (28.3)
First degree	601 (60.0)
Masters’ degree	88 (8.8)
PhD	8 (0.8)
Missing	21 (2.1)
Profession	
Doctor	203 (20.26)
Pharmacist	159 (18.1)
Medical laboratory scientist	161 (18.3)
Nurse	461 (46.0)
Others	12 (1.2
Missing	6 (0.6)
Genotype	
I don't know	68 (6.8)
AA	652 (65.1)
AS	212 (21.2)
AC	33 (3.3)
SS	6 (0.6)
SC	2 (0.2)
Missing	29 (2.9)

### Funding and support for sickle cell disease

**Figure 1:**
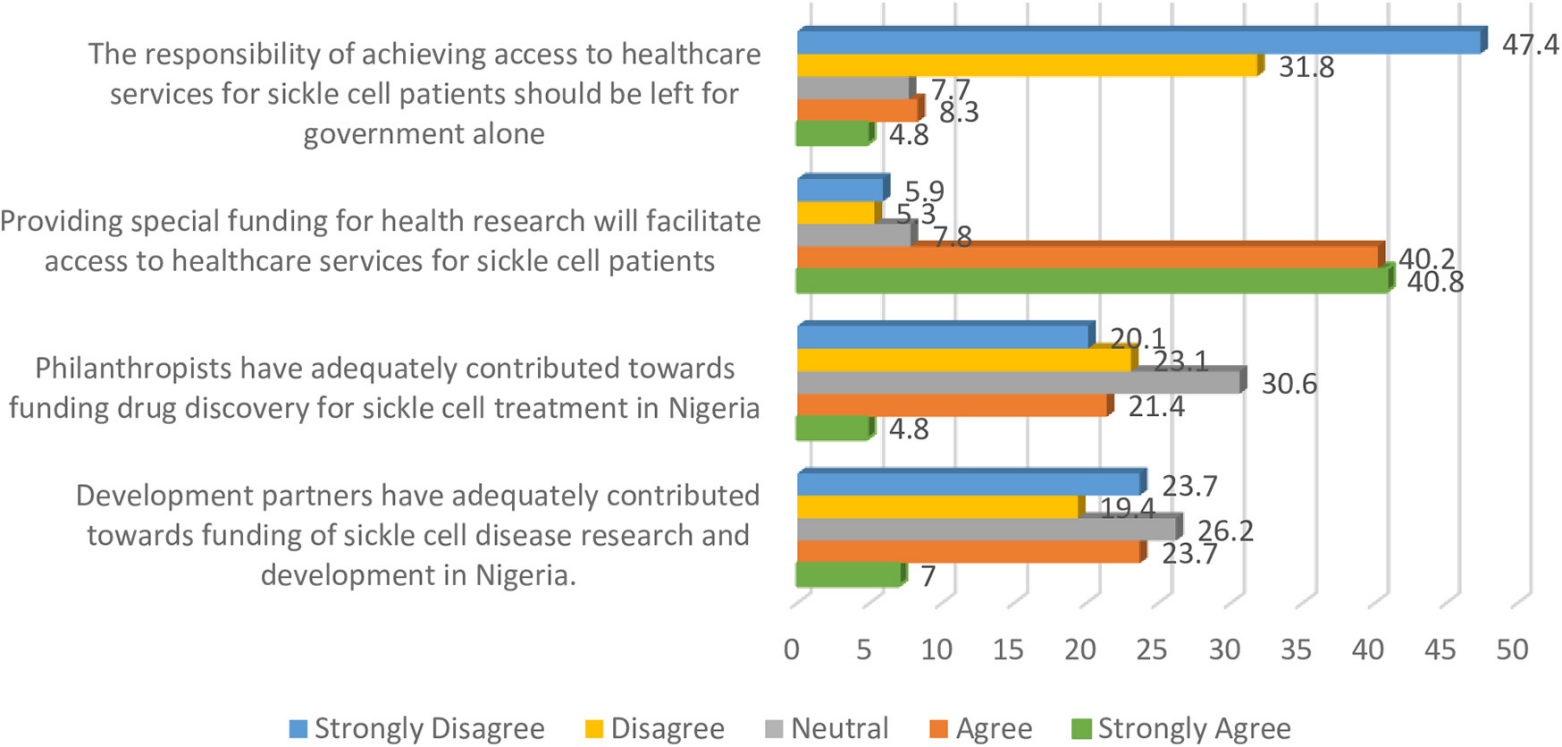
Funding and support for sickle cell.

Only about one-third of the respondents (30.7%) indicated that development partners have adequately contributed to funding sickle cell research in Nigeria. A strong majority of the sample (81%) indicated that special funding provision for health research could facilitate access to healthcare services for sickle cell patients. A similar proportion (79.2%) disagreed that the responsibility of achieving access to healthcare services for sickle cell patients should be left to the government alone. Further details on funding and support for sickle disease are presented in Figure 1.

Figure 1 summarises respondents' perceptions regarding funding contributions and the role of various stakeholders in supporting healthcare services for sickle cell disease.

### Primary healthcare involvement in management of sickle cell disease

**Figure 2:**
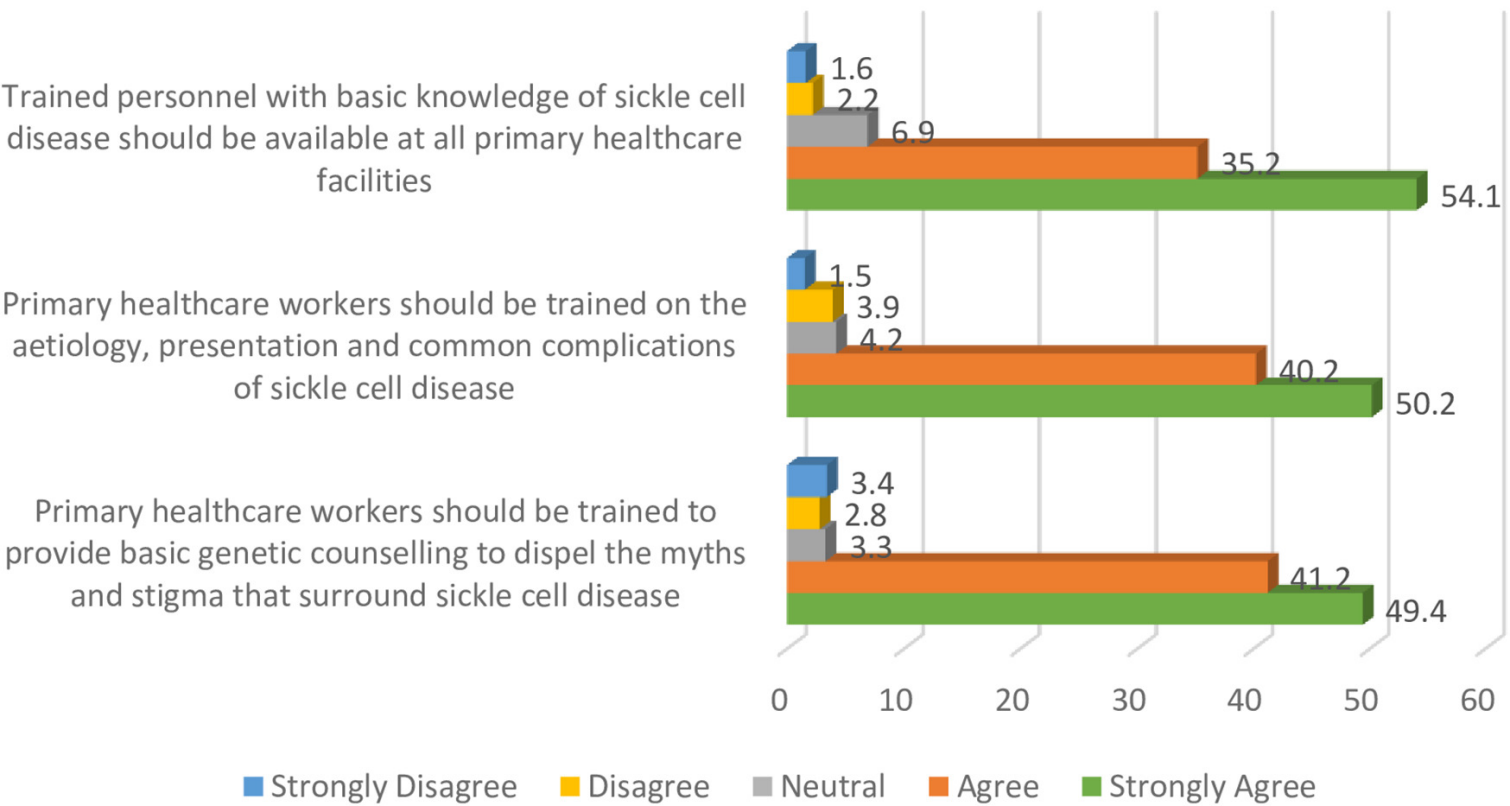
Improving access to healthcare services through strengthening of primary health centres.

A strong majority of the participants (89.3%) indicated that trained personnel with knowledge of sickle cell disease should be available at all primary healthcare centres, and a similar proportion (90.4%) were of the view that primary healthcare workers should be trained on presentations and common complications associated with the condition. Other relevant details are presented in Figure 2.

The findings presented in Figure 2 indicate that 90.6% of the participants were of the perspective that primary healthcare workers should be trained to provide basic genetic counselling to dispel myths and stigma associated with the disease.

### Research focusing on sickle cell disease

**Figure 3:**
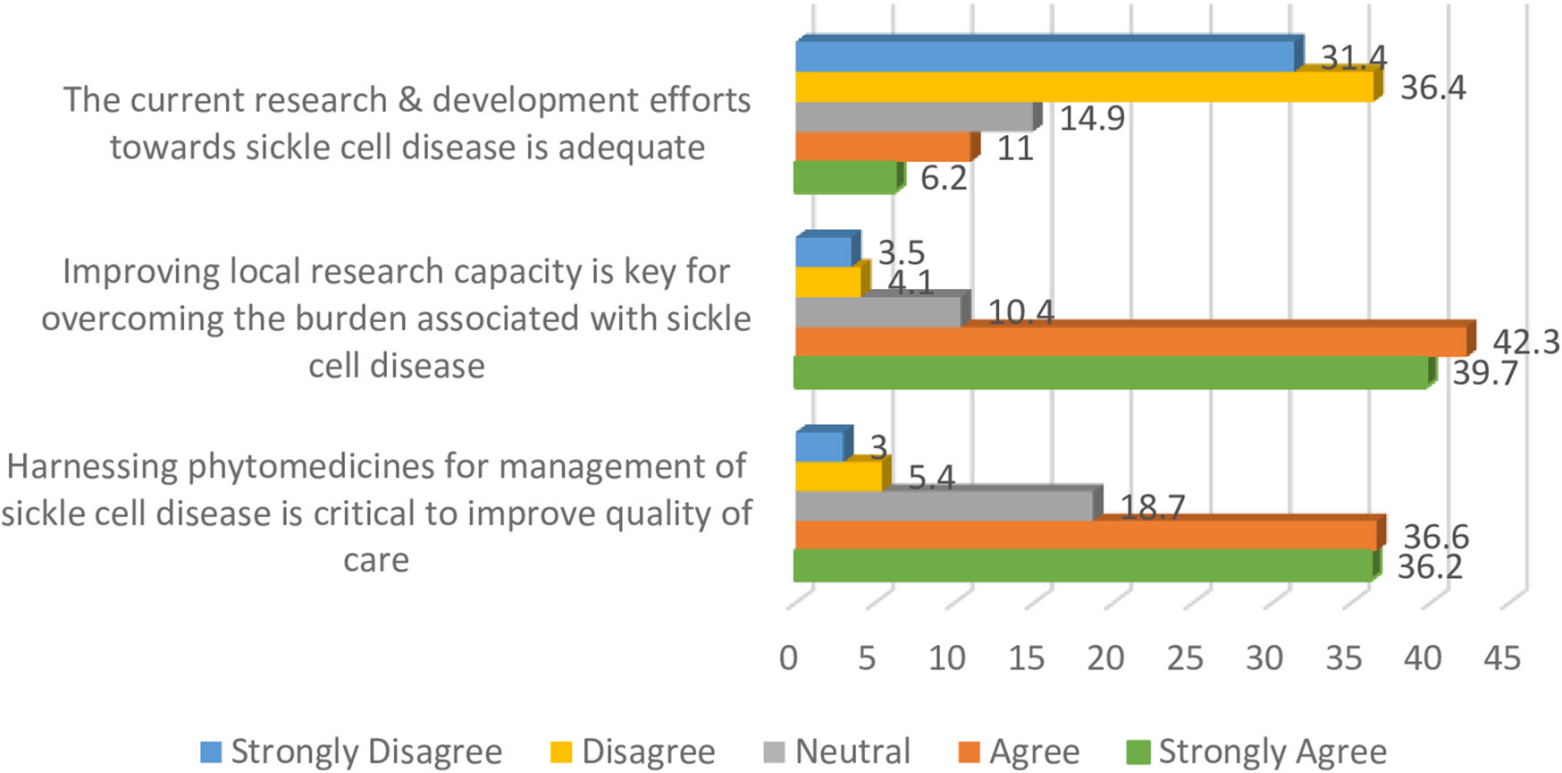
Research and development activities in relation to sickle cell disease.

Respondents in this study were dissatisfied with the research and development activities focused on sickle cell disease, as indicated by 67.8% of the study population. Participants' further perceptions on the inadequacy of the efforts in this area are presented in Figure 3.

Participants agreed that improving local research capacity was key to overcoming the burden associated with sickle cell disease, whilst also supporting the need to harness phytomedicines for the management of the condition.

Collectively, only a quarter of the participants (24.1%) rated the current research and development efforts towards sickle cell disease as good and excellent, whilst others gave fair and poor ratings.

**Figure 4:**
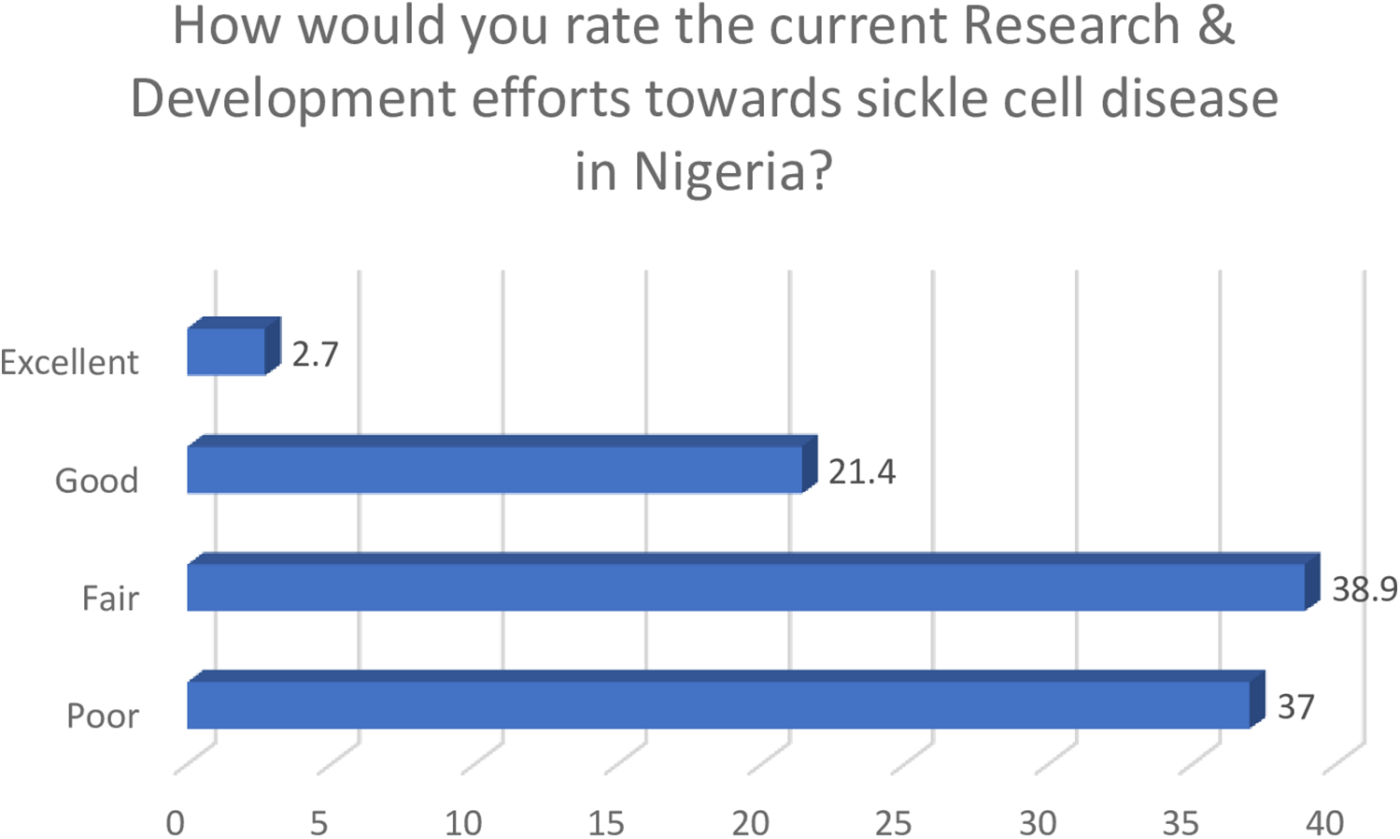
Research and development rating.

Figure 4 shows participants' ratings of current research and development efforts on sickle cell disease, with only 24.1% describing them as good or excellent.

### Association between demography, funding, and support

In addition to the descriptive statistical analyses undertaken, Chi-square tests were carried out. The association between participants' views with the statement “providing special funding for health research will facilitate access to healthcare services for sickle cell patients” was significantly different across genotype (*p* < 0.001) and highest educational level (*p* = 0.011). All the healthcare professionals (100%) who indicated being SC disagreed with the statement compared to those who were AA (7.6%), AS (16.1%), SS (33.4%), and those who did not know their genotype (24.2%). Also, the findings indicate that the majority of the participants with diplomas (75.5%), first degrees (84.2%), and master's degrees (79.3%) agreed with the statement as compared to only 50% of doctoral degree holders who shared similar opinions. This result was statistically significant (*p* = 0.011). Similarly, none of the sickle cell participants were of the opinion that the responsibility of achieving access to healthcare services for sickle cell patients should be left for the government alone (*p* = 0.007). Also, a large percentage of participants with diplomas (71.3%), first-degree (83.8%), and master's degrees (78.5%) disagreed with the view that the responsibility of achieving access to healthcare services for sickle cell disease patients should rest solely with the government. In contrast, only 25% of PhD holders shared this opinion. This outcome was also statistically significant (*p* < 0.001).

## Discussion

From the perspective of healthcare professionals, philanthropists, the private sector, and other non-governmental organisations have not adequately contributed to the funding of sickle cell disease management as well as research. Interestingly, however, the majority of the participants disagreed that the responsibility of achieving access to healthcare for sickle cell disease patients should be left to the government alone. These findings suggest that the burden of sickle cell disease can be significantly reduced if charitable foundations, philanthropists, private sector actors, and other non-profit organisations in Nigeria contribute to research endowments. Comparable evidence in developed countries imply that this approach is more sustainable and impactful. In fact about half of the health research activities in such settings are funded by public and charitable organisations (35, 36). These agencies play important roles in pharmaceutical product development, particularly in areas that are not traditionally viewed as profitable, such as neglected tropical diseases (37).

A strong majority of participants in this study were of the view that providing special funding for research can facilitate access to healthcare services for persons with sickle cell disease, a finding that aligns with previous reports relating to Medicines' Security and access to high-quality healthcare (38). However, only half of the participants with doctoral degrees agreed with this stance. a result that, while statistically significant, must be interpreted in light of the small number of PhD holders in the study. An in-depth examination of associations between demographic and professional variables may help explain this: respondents with doctoral qualifications were less likely to endorse special funding as a facilitator of access, possibly reflecting their academic orientation and critical appraisal of systemic funding challenges or greater awareness of structural research barriers. Conversely, professionals with diplomas, first-degree, or master's level qualifications are often more engaged in frontline clinical roles, may perceive direct benefits of targeted funding. Differences may also exist across professions: clinicians directly managing sickle cell disease cases (e.g., nurses, laboratory scientists) might prioritise interventions differently than policy-oriented practitioners. Respondents with sickle cell disease were of the opinion that the responsibility of achieving access to healthcare services for sickle cell patients should not be left to the Government alone. Gender-based or regional differences may further shape perceptions due to diverse professional experiences and cultural expectations in healthcare delivery. These hypotheses should be explored in future qualitative or stratified analyses to tailor interventions effectively to different stakeholder groups.

Furthermore, participants in this study supported the need for the inclusion and training of primary healthcare workers in the management of sickle cell disease, and this appears to be in line with a previous study reported in India (24). In addition, participants indicated that there was a need to build the capacity of healthcare providers at primary health centres for basic genetic counselling. In Nigeria, care for sickle cell disease has been largely confined to tertiary healthcare facilities with few dedicated sickle cell clinics (23), and a previous study undertaken in this setting showed that primary healthcare workers had poor knowledge of sickle cell disease diagnosis and crisis prevention (22). Findings from this study therefore provide contemporary insights into some key areas that can be considered in developing contextual strategies to reduce the burden of sickle cell disease.

Findings from this study identified current research and development efforts towards sickle cell disease as inadequate, and collectively, three-quarters of the respondents rated activities in this area as suboptimal. These findings suggest desperate and urgent reforms that can underpin comprehensive research activities for sickle cell disease. It is important for policymakers and the government to continuously explore novel mechanisms that can improve research in this area, as Nigeria reportedly bears the largest burden of the disease globally. Intensifying and prioritising research activities towards sickle cell disease can play a critical role in improving access to healthcare services for people suffering from these conditions (11, 39). Participants were of the opinion that improving local research capacity was key to overcoming the burden of sickle cell disease, and interestingly, they supported the need to harness phytomedicines for the management of the disease as a means of improving quality of care. This position is similar to previous findings that identified the need to articulate research activities in areas related to medicinal plants and other natural resources as a key strategy for improving access to healthcare services (40, 41). This novel finding from our study therefore supports the need for a paradigm shift in the search for solutions for sickle cell disease, as the only available phytomedicine for the management of the disease was developed in Nigeria via this approach. This product has gained international relevance, making it widely accepted in many countries (42, 43). Whilst this study provides evidence that validates the need for critical attention to phytomedicinal research the output also represents an opportunity to develop a robust strategy that can appropriately harness these long-neglected opportunities. When designing strategies to improve control of sickle cell disease, it is important to set clear and measurable short-term, as well as long-term goals. Likewise, regular monitoring and evaluation are necessary to ensure sustainability of such interventions. Gyamfi et al. (44), reported specific aspects of care for sickle cell patients that need to be considered when designing evidence-based interventions for the condition. These include education of patients and care-givers, supportive care for pain and crises, management of infections, as well as the use of disease modifying drugs and nutritional supplements.

This study provides insights from the perspective of healthcare practitioners in Nigeria on access to healthcare for people with sickle cell disease. The approach adopted for this study enabled the collection and collation of broad perceptions of healthcare professionals' views and experiences regarding the disease. Despite these strengths, several limitations warrant consideration. The study did not explore some therapeutic components of sickle cell disease management that are often integrated into primary healthcare services, such as penicillin prophylaxis, immunisation, hydroxyurea therapy, and newborn screening programmes. This was primarily due to the study's focus on broader healthcare access and policy-related perspectives. Additionally, the small number of PhD-qualified respondents constrained optimal subgroup analyses. Self-reported data from facilities already managing sickle cell disease may have introduced social desirability and selection biases while excluding perspectives from settings without formal sickle cell disease services. Although all zones were included, practitioners in remote or underserved areas may still be underrepresented, affecting generalisability. The cross-sectional design precludes causal inferences, and the survey while validated might not capture all nuanced institutional or patient-level barriers. Further longitudinal and qualitative studies are needed to deepen understanding and confirm these associations. Moreover, while these findings are directly relevant to Nigeria and similar low-resource contexts, caution is advised in generalising to regions with different healthcare systems, resource profiles, or disease burdens. Further studies in this area are recommended to provide deeper insights into the emergent findings.

## Conclusion

The novel insights revealed in this study emerged from the perspectives of healthcare professionals regarding how access to care can be enhanced for people with sickle cell disease. To improve access to healthcare services for sickle cell disease patients, proactive yet contextual policies need to be initiated by the government alongside stakeholders, with the aim of attracting funding and support from charities, philanthropists, the private sector, and non-profit organisations.

The findings from this study revealed that the inclusion of primary healthcare centres in the sickle cell management structure could improve access for people with sickle cell disease. In addition, regular capacity-building training for staff of primary healthcare centres can empower them with the necessary knowledge and skills to provide premarital counselling for patients seeking healthcare services at the grassroots level.

Given the high prevalence of sickle cell disease in this region, there is an urgent need for the government to scale up research activities in relation to this condition. It is also important to develop a robust all-inclusive framework aimed at preventing and reducing the disease burden. The output of this intervention also highlights the importance of prioritising the development of local research capacity, especially in relation to harnessing the significant potential of phytomedicines for the prevention, management, and treatment of diseases.

The findings reported in this study are novel, as little empirical work has been undertaken in this area, especially for the contextual setting. These outcomes can consequently guide governments and relevant stakeholders in developing robust strategies to improve access to healthcare services for sickle cell disease in Nigeria and other countries with similarly high burden of the condition.

## Data Availability

The raw data supporting the conclusions of this article will be made available by the corresponding authors on reasonable request.
