# Supplementary material for: Improving access to healthcare services for sickle cell disease patients in Nigeria: perspectives and views of healthcare professionals
Source: Front Health Serv. 2025 Aug 25;5:1466299. doi: 10.3389/frhs.2025.1466299 (PMC12415052; doi:10.3389/frhs.2025.1466299)
Supplement: Supplementary file 1 [file Table1.docx]

**NATIONAL INSTITUTE FOR PHARMACEUTICAL RESEARCH AND DEVELOPMENT (NIPRD), IDU ABUJA**

**Improving Access to Healthcare Services for Sickle Cell Disease Patients in Nigeria: Perspectives and Views of Healthcare Professionals**

**Introduction**

Sickle cell disease (SCD) is a group of blood disorders typically inherited from parents. This condition can lead to multiple organ dysfunctions with several clinical consequences. This questionnaire aims at understanding issues surrounding the management of sickle cell disease in Nigeria as well as articulating strategies to improve quality of care for affected individuals. Please fill the questionnaire by ticking (√) the most appropriate option(s). Your responses will be treated confidentially.

**Demographic Data**

1. Gender

| Male | Female |
| --- | --- |

2. Age

| ≤30 | 31 - 40 | 41-50 | 51 and above |
| --- | --- | --- | --- |

3. Highest Educational Level

| Diploma | First degree/HND | Master’s degree | PhD |
| --- | --- | --- | --- |

4. Profession

| Doctor | Pharmacist | Medical Lab. Sci. | Nurse | Others, please specify ...................... |
| --- | --- | --- | --- | --- |

5. Genotype

| I don’t Know | AA | AS | AC | SS | SC |
| --- | --- | --- | --- | --- | --- |

**For the following statements, please indicate your level of agreement: SD (Strongly Disagree), D (Disagree), N (Neutral), A (Agree), SA (Strongly Agree).**

**Funding and Support for Sickle Cell Disease**

| **SN** | **Statement** | **SD** | **D** | **N** | **A** | **SA** |
| --- | --- | --- | --- | --- | --- | --- |
| 6 | Development partners have adequately contributed towards funding of sickle cell disease research and development in Nigeria. |  |  |  |  |  |
| 7 | Philanthropists have adequately contributed towards funding drug discovery for sickle cell treatment in Nigeria. |  |  |  |  |  |
| 8 | Charitable foundations have adequately contributed towards funding of sickle cell disease treatment and management in Nigeria. |  |  |  |  |  |
| 9 | Providing special funding for health research will facilitate access to healthcare services for sickle cell patients. |  |  |  |  |  |
| 10 | The responsibility of achieving access to healthcare services for sickle cell patients should be left for the government alone. |  |  |  |  |  |

**Improving Access to Healthcare**

| **SN** | **Statement** | **SD** | **D** | **N** | **A** | **SA** |
| --- | --- | --- | --- | --- | --- | --- |
| 11 | Primary healthcare workers should be trained to provide basic genetic counselling to dispel the myths and stigma that surround sickle cell disease. |  |  |  |  |  |
| 12 | Primary healthcare workers should be trained in the aetiology, presentation and common complications of sickle cell disease. |  |  |  |  |  |
| 13 | Trained personnel with basic knowledge of sickle cell disease should be available at all primary healthcare facilities. |  |  |  |  |  |
| 14 | Psychosocial support is key in management of sickle cell disease. |  |  |  |  |  |
| 15 | Harnessing phytomedicines for management of sickle cell disease is critical to improve quality of care. |  |  |  |  |  |
| 16 | Improving local research capacity is key for overcoming the burden associated with sickle cell disease. |  |  |  |  |  |
| 17 | The current research & development efforts towards sickle cell disease is adequate. |  |  |  |  |  |
| 18 | Research and development towards sickle cell disease treatment has been adequately prioritized in Nigeria. |  |  |  |  |  |

19. How would you rate the current health interventions of sickle cell disease in Nigeria?

(a) Poor [ ] (b) Fair [ ] (c) Good [ ] (d) Excellent [ ]

20. How would you rate the current Research & Development efforts towards sickle cell disease in Nigeria?

(a) Poor [ ] (b) Fair [ ] (c) Good [ ] (d) Excellent [ ]

**Thank you for taking your time to complete this questionnaire**
